# Exploring the Acceptability of Post-bariatric Nutritional-Behavioral and Supervised Exercise Intervention (BARI-LIFESTYLE): A Mixed Methods Evaluation

**DOI:** 10.1007/s11695-025-07927-0

**Published:** 2025-05-31

**Authors:** Friedrich C. Jassil, Raissa Hamelmann, Adrian Brown, Alisia Carnemolla, Helen Kingett, Jacqueline Doyle, Amy Kirk, Neville Lewis, Gemma Montagut, Parastou Marvasti, Kusuma Chaiyasoot, Roxanna Zakeri, Jessica Mok, Kalpana Devalia, Chetan Parmar, Janine Makaronidis, Rachel L. Batterham

**Affiliations:** 1 Centre for Obesity Research, University College London, London, United Kingdom; 2 Service of Endocrinology, Diabetology, Nutrition and Therapeutic Education, Department of Medicine, Geneva University Hospitals, Geneva, Switzerland; 3 Bariatric Centre for Weight Management and Metabolic Surgery, University College London Hospitals NHS Trust, London, United Kingdom; 4 Division of Women’s Health, University College London Hospitals NHS Trust, London, United Kingdom; 5 The Hatter Cardiovascular Institute, Institute of Cardiovascular Science, University College London, London, United Kingdom; 6 Division of Nutrition, Department of Medicine, Faculty of Medicine Siriraj Hospital, Mahidol University, Bangkok, Thailand; 7 Bariatric Surgery Department, Homerton University Hospital NHS Trust, London, United Kingdom; 8 Department of Surgery, Whittington Health NHS Trust, London, United Kingdom; 9 University College London, London, United Kingdom; 10 National Institute for Health Research, UCLH Biomedical Research Centre, London, United Kingdom; 11 Diabetes Centre, Faculty of Medicine, University of Geneva, Geneva, Switzerland

**Keywords:** BARI-LIFESTYLE, MBS, Obesity, Diet, Exercise, Qualitative

## Abstract

**Background:**

The BARI-LIFESTYLE trial explored the impacts of a combined nutritional-behavioral tele-counselling and supervised exercise intervention in the first year following bariatric surgery. While the program did not elicit additional weight loss or improvements in health outcomes, evaluating its acceptability remains critical to refining future research and intervention design.

**Methods:**

A mixed-methods approach was employed. First, the acceptability of the intervention program was determined through randomization refusal rate, dropout rate, intervention refusal rate, and attendance rate. Data from the self-reported exit questionnaire completed at the final study visit were analyzed using descriptive statistics, and free-text responses were examined using a content analysis approach.

**Results:**

A total of 79 participants (74.7% female; mean ± SD age 44.8 ± 10.8 years; mean BMI 42.1 ± 5.8 kg/m^2^) were randomly assigned to the BARI-LIFESTYLE program. The randomization refusal rate was 2%. The tele-counselling achieved high acceptability, as evidenced by a low refusal rate (1.3%), and high attendance (79%), with 96.8% reporting the sessions as useful. Qualitative data further highlighted its role in supporting post-bariatric surgery lifestyle adaptation. In contrast, the supervised exercise program exhibited moderate acceptability, with a higher refusal rate (21.6%) and an attendance rate of 72.4%. Despite this, 98.1% of regular attendees found the sessions beneficial, particularly for addressing physical and psychological barriers to exercise. Key barriers to participation in both interventions included competing demands and scheduling conflicts. To improve the acceptability of future interventions, recommendations include the integration of mobile technology, increasing the frequency of tele-counselling sessions, enhancing accessibility to exercise classes, and providing personalized exercise programs.

**Conclusions:**

Participant-reported outcomes suggest that the BARI-LIFESTYLE program provided holistic support, addressing diet, exercise, social, and psychological aspects of life after bariatric surgery.

**Supplementary Information:**

The online version contains supplementary material available at 10.1007/s11695-025-07927-0.

## Introduction

Metabolic bariatric surgery (MBS) leads to substantial weight loss and the improvement or resolution of obesity-associated medical problems, ultimately enhancing quality of life and reducing overall mortality [1]. However, MBS outcomes vary significantly among individuals, with one in four patients experiencing less than 20% weight loss [2]. One key contributing factor is the difficulty in adapting to the necessary lifestyle changes after surgery [3, 4]. These challenges include increased calorie intake over time, insufficient protein consumption, poor adherence to vitamin and mineral supplementation, and low levels of physical activity, with a high amount of time spent in sedentary behavior [3, 4]. In response, the UK National Institute for Health and Care Excellence (NICE) has recommended research to assess whether adjunctive post-surgical lifestyle intervention programs can improve weight loss outcomes and the overall health benefits of MBS [5].

To address this research gap, we developed and implemented the BARI-LIFESTYLE intervention program, which integrated post-surgery nutritional-behavioral tele-counselling with supervised exercise classes. Its efficacy was evaluated using a randomized controlled study design [6]. Our findings revealed that this adjunctive lifestyle program, initiated immediately after MBS, did not yield additional weight loss or health outcomes at the 12-month post-surgery follow-up. However, assessing its acceptability remains essential for refining future research and intervention strategies. Additionally, acceptability data, including participant-reported outcome measures, can help identify factors influencing enrolment, adherence, and retention in the program—key determinants that may impact overall efficacy [7]. Such insights are crucial for shaping future lifestyle intervention research for patients undergoing MBS. Therefore, this study aims to evaluate the engagement, acceptability, usability, and satisfaction associated with the BARI-LIFESTYLE intervention program.

## Materials and Methods

### Study Design and Participants

BARI-LIFESTYLE was a two-arm, parallel-group, single-blind, multisite randomized controlled trial (RCT) embedded within an observational cohort study [6, 8]. This RCT included 153 patients undergoing Roux-en-Y gastric bypass, one-anastomosis gastric bypass, or sleeve gastrectomy across three National Health Service (NHS) bariatric centers in London, UK. Of the 153 participants in the observational cohort, 79 were randomly assigned to the intervention group, which received the BARI-LIFESTYLE program alongside standard post-surgery care. The study protocol, including the primary and secondary outcomes of the BARI-LIFESTYLE trial, has been previously published [6, 8–10].

### The BARI-LIFESTYLE Intervention Program

The program consisted of 17 tele-counselling sessions in the first year of surgery that commenced in the first week following surgery. The tele-counsellors were bariatric dietitians trained in behavioral psychological techniques. Participants were provided with a tele-counselling booklet containing dietary recommendations with diaries to self-report their food intake, supplements intake, step count, and body weight (Supplementary S1). The information reported by participants in the diaries was used by the tele-counsellor to guide and individualize the content of each session. At 3 months post-surgery, participants were enrolled in a once-weekly individually tailored supervised exercise program for 12 weeks. Each session lasted for 60 min that combined both aerobic and resistance training. Participants were provided with an exercise booklet containing a weekly exercise log for 12 weeks (Supplementary S2). The classes were delivered by exercise therapists at the hospital gym. However, during the COVID-19 pandemic, the classes were delivered remotely via Zoom, and participants’ views and experiences on the tele-exercise have been published separately [9].

### Outcome Measures

All tele-counselling and exercise sessions, including reasons for refusal to take part in the intervention and non-attendance, were recorded in the trial database by the tele-counsellors and exercise therapists. In addition, all participants who received the lifestyle intervention were asked to complete a self-reported exit questionnaire, developed by the research team, during the final study visit at 12-month post-surgery follow-up (Supplementary S3). This questionnaire asked participants to rate the quality of both the tele-counselling sessions and supervised exercise classes and captured their views on the contents, length, homework, and materials provided in the program. The questionnaires also covered self-reported barriers and facilitators that contributed to their compliance with the program. Open-ended questions with free-text comments were included to elicit additional information from participants to support the quantitative findings, enabling the identification of aspects that may influence the interventions’ acceptability.

### Data Analysis

Acceptability refers to the suitability of the interventions from the participants’ perspective and is assessed through the following [7]:

1. Randomization refusal rate (%) = 100/number of participants randomized × number of participants who refused randomization
2. Dropout rate (%) = 100/number of participants allocated to the intervention × number of participants who discontinued the intervention
3. Intervention refusal rate (%) = 100/(number of participants enrolled + number of participants who declined to participate in the tele-counselling or supervised exercise) × number of participants who declined to participate in the tele-counselling or supervised exercise
4. Attendance rate (%) = number of completed sessions (tele-counselling or supervised exercise)/the total sessions prescribed (17 sessions for tele-counselling or 12 sessions for supervised exercise) × 100

Unless otherwise stated, continuous data are presented as means and standard deviations, while categorical data are presented as counts and percentages.

The qualitative analysis involved reading each participant’s free text responses in detail to support the quantitative findings. Whereas the free text responses on the overall overview of the BARI-LIFESTYLE program were examined using a content analysis approach [11]. Two researchers (FCJ and RH) independently read and coded the free-text responses and discussed their preliminary codes and refined them through an iterative process. Next, FCJ and RH extracted the codes that shared similar ideas and concepts that represented broader-level categories. These categories were organized to generate themes based on counting the frequency of codes. Specific quotations were then extracted to illustrate the themes. FCJ is a dietitian with training and experience in conducting and analyzing qualitative data, while RH is a medical practitioner undertaking a postgraduate master’s dissertation in qualitative research. FCJ conceptualized, executed, and led the RCT, whereas RH was not involved in the wider trial.

## Results

This analysis included 79 participants (74.7% female) with a mean ± SD age of 44.8 ± 10.8 years and a mean BMI of 42.1 ± 5.8 kg/m^2^ that were randomized to the intervention group (Table 1). Of these, a total of 63 (82.9%) participants who received the lifestyle intervention completed the self-reported exit questionnaire at the end of the trial.

**Table 1:** Baseline characteristics of the intervention group

Participant characteristics (*n* = 79)	
Age (years)	44.8 ± 10.8
Sex, *n* (%)	
Male	20 (25.3)
Female	59 (74.7)
Menopause, *n* (%)	15 (25.4)
Weight (kg)	119.1 ± 18.2
Height (m)	1.68 ± 0.08
Body mass index (kg/m^2^)	42.1 ± 5.8
Type of surgery, *n* (%)	
Roux-en-Y gastric bypass	24 (30.4)
One anastomosis gastric bypass	13 (16.5)
Sleeve gastrectomy	42 (53.1)
Surgery center, *n* (%)	
University College London Hospitals	37 (46.8)
Whittington	21 (26.6)
Homerton	21 (26.6)
Ethnicity, *n* (%)	
White/White British	43 (54.4)
Mixed race	4 (5.1)
Asian/Asian British	7 (8.9)
Black/Black British	19 (24.0)
Other ethnicity	6 (7.6)
Education level, *n* (%)	
No qualification	6 (7.6)
GCSE/O equivalent	14 (17.7)
A level equivalent	16 (20.3)
University degree	24 (30.3)
Higher degree	16 (20.3)
Other	3 (3.8)
Marital status, *n* (%)	
Single	23 (29.1)
Married/lives with a partner/civil partnership	45 (57.0)
Separated/divorced	9 (11.4)
Widow	2 (2.5)
Employment status, *n* (%)	
Employed	55 (69.6)
Unemployed	14 (17.7)
Others	10 (12.7)
Obesity-associated medical problems, *n* (%)	
Type 2 diabetes	19 (24.1)
Hypertension	31 (39.2)
Hyperlipidemia	12 (15.2)
Obstructive sleep apnea	27 (34.2)

Abbreviations: *GCSE* General Certificate of Secondary Education, *kg* kilograms, *kg/m*^2^ kilograms per meter squared, *m* meters, *n* number, %, percentage

### Randomization Refusal Rate, Dropout Rate, Intervention Refusal Rate, and Attendance Rate

The randomization refusal rate was 2% (*n* = 3). Of 79 participants randomized to the intervention group, three participants did not consent to the BARI-LIFESTYLE program. The reasons for randomization refusal were work demands (*n* = 1) and personal reasons (*n* = 1), with the third participant not stating a reason (*n* = 1). The dropout rate of the intervention program was 5.3% (*n* = 4). The reasons for dropout were due to pregnancy (*n* = 2) and personal reasons (*n* = 2).

Of 76 participants allocated to the intervention group, one participant declined to participate in the tele-counselling sessions due to personal reasons, hence a 1.3% refusal rate. The attendance rate for the tele-counselling sessions was 79%. The most commonly reported reasons for rescheduled or cancelled sessions were family commitments, followed by forgetting about the tele-counselling appointment, being away on holiday, healthcare appointments, and sickness.

For the supervised exercise program, 21 participants declined to participate, resulting in a 21.6% refusal rate (*n* = 21). Reasons for refusal were work demands (*n* = 5), family commitments (*n* = 4), preferring to do their own exercises (*n* = 3), personal reasons (*n* = 3), inconvenient gym location and exercise schedule (*n* = 2), and did not state (*n* = 4). The attendance rate for the supervised exercise classes was 72.4%. The most commonly reported reasons for non-attendance were mainly family commitments, followed by sickness and logistical issues (e.g., the gym location being too far from home, resulting in long travel times).

### Exit Questionnaire

Detailed results from the exit questionnaire are presented in Supplementary S4. Overall, 96.8% of participants felt that the tele-counselling sessions were useful. For instance, this participant commented, “The tele-counselling was imperative to my success” (Participant 002 [P002]). Participants reported that the sessions provided them with the opportunity for “discussing diet and health tips” (P065), with their assigned tele-counsellor. Specifically, topics such as food portions, protein-rich food, meal planning, and meal preparation were useful to them. Some of the participants also opined that the sessions had offered them with “coping mechanism[s]” associated with their eating habits post-surgery. For example, one participant wrote, “[Tele-counsellor name] was very interactive and was helpful at times when my appetite was not good, suggesting alternatives” (P036)*.* Whereas other participants mentioned the sessions helped them “dealing with food temptations” (P077) and “helped with snacking and hunger pangs” (P028). During the sessions, participants also had the opportunity to raise some common post-surgery issues that needed explanations and solutions from the tele-counsellors such as constipation, nausea, and hair loss.

Most participants (88.9%) were satisfied with the quality of the tele-counselling booklet and materials provided. In addition, 88.9% of participants found the materials provided to be easy to use, while 90.5% were satisfied with the amount of help provided by the tele-counsellors on how to complete the diaries. This participant further stated, “It was helpful to knowing what you actually eat, and I could always refer back to them!” (P036). However, 7.9% of participants felt the materials provided were difficult to use. For example, these participants described the diary as “…too time consuming and inconvenient to fill” (P139) whereas this participant said, “I tend to forget to fill in the diaries” (P054). Other participants also expressed that the diary was “a little confusing” (P151) and had “not enough space to write all foods” (P126). Therefore, as a solution, three participants suggested using smartphone applications or an online diary, which were perceived to be easier than the paper-based diary: “An app to record data would be ideal than paper copies” (P062).

In terms of the call length, 84.1% of participants were satisfied, though one participant felt the length was too short. Of all participants, 92.1% did not find any difficulty in booking the tele-counselling session with their tele-counsellor, with this participant said, “They[the tele-counsellors] were so accommodating” (P064). Nevertheless, one participant found it challenging to have the session during working hours, “It all depends on if you are working and being allowed to speak with your tele-counsellor. It’s all work permitted” (P127). Several participants suggested a few strategies to improve the tele-counselling sessions such as offering more frequent sessions during the maintenance phase between 6 and 12 months post-surgery. This participant felt, “The last few months is the hardest as appetite comes back so it should be monthly instead of three months” (P016). Furthermore, some participants preferred to have the same tele-counsellor throughout the program, and two participants suggested considering the use of video calls in the future, “Consider video calls—may not be practical or possible to all patients but some would find it beneficial” (P148).

Out of 52 participants who completed the exercise section of the questionnaire, 98.1% found the tailored supervised exercise program useful. One participant commented, “Enjoyed the choice and range of exercises” (P062). In particular, many participants enjoyed the resistance training using gym equipment, resistance bands, and bodyweight exercises. For example, one participant appreciated “Learning how to use the exercise machines” (P045). Exercises such as the leg press and weight training targeting the arms were the most frequently mentioned as enjoyable. In contrast, exercises like press-ups, planks, lunges, sit-ups, squats, and other floor-based exercises were the least enjoyable, as participants found them challenging. Some participants noted that pre-existing obesity-associated medical problems limited their ability to perform these exercises. As one participant explained, “Pre-existing injuries delayed some exercise but did get easier” (P049). Another participant expressed appreciation for the exercise therapist’s support in tailoring exercises to their functional limitations, stating, “I had shoulder problems but [therapist name] helped me adapt in real-time” (P117).

In terms of aerobic training, exercising on the treadmill and the stationary bicycle were among the participants’ favorites. However, cardio machines such as the climbmill and cross trainer were found to be challenging. As one participant noted, “The climbmill is difficult because of my osteoarthritis” (P074). Additionally, another participant felt that “the use of cardio equipment alone was monotonous” (P056). One particular aspect that some participants enjoyed during the supervised exercise classes was the social element. As one participant shared, “The weekly group exercise was fun and interactive. Attending regularly give focus on the need and benefits of exercising” (P143).

Regarding the length of exercise classes, 69.2% of participants were satisfied, while 13.5% felt the classes were too short, and 1.9% perceived them as too long. One participant commented, “The one hour session often stretched to approximately 1½ hours which was good” (P015). Overall, 92.3% of participants were satisfied with the quality of time, and 88.5% were satisfied with the amount of time they spent with the exercise therapist. However, some participants expressed dissatisfaction, stating, “I hardly saw the therapist” (P045) and “I would have liked longer” (P097). Participants highlighted several key qualities of the exercise therapists, including professionalism, attentiveness, supportiveness, and care. As one participant noted, the therapist “knows our limits and pushed us every week” (P016). Conversely, an exercise routine that was not personalized to participants’ fitness levels was found to be demotivating. For example, one participant was dissatisfied with the quality of exercise received, commenting, “Had a generic basic exercise plan” (P074). Overall, 94.2% were satisfied with the exercise booklet provided.

In terms of the exercise schedule and location, six participants (11.6%) found it difficult to choose a convenient exercise session due to logistical issues. One of the participants commented, “The only area I found a struggle was travelling to London each week” (P062). To address this, participants suggested that future programs offer more options for gym locations and time slots, including additional evening and weekend sessions. As one participant noted, “It’s difficult for people who work Mon-Fri” (P015). For the evening session, a start time slightly later than 5:30 pm was preferred. Additionally, seven participants recommended extending the exercise program beyond 12 weeks. For example, one participant remarked, “More sessions and perhaps on the session after 12-month post-surgery to keep you active” (P028).

Overall, participants highlighted multiple benefits of the BARI-LIFESTYLE program and suggested a few recommendations for future programs, as summarized in Table 2. A majority of participants (40.2%) emphasized that the program provided ongoing support and guidance, which they found invaluable for both mental and practical aspects of their weight loss journey. Many noted that support was readily available whenever they had questions or needed advice. Additionally, 22.1% of participants reported gaining essential knowledge about the post-bariatric surgery diet and exercise, empowering them to make informed food choices and identify exercise routines they felt comfortable with. Supervised exercise sessions were particularly impactful. Approximately 20.8% of participants perceived that the structured sessions had helped them adopt regular, intense physical activity sooner than they would have independently, thereby boosting their confidence and reducing fears around exercise. Some participants (9.1%) also highlighted how the program assisted them in coping with the challenges they faced following bariatric surgery, not only in terms of diet and exercise but also on how to adjust themselves within society. In addition, participants (7.8%) also emphasized how the program kept them accountable during difficult phases, stating it helped them “refocus” during “bad months” and stay on track with their weight loss goals. Finally, the most common suggestions for future programs include extending the duration beyond 1 year post-surgery (77.8%) and offering the program during the pre-surgery period (22.2%).

**Table 2:** The most frequently mentioned benefits of the BARI-LIFESTYLE program and recommendations for future program

**Benefits**	***n*** (%)	**Quotations**
Constant support	31 (40.2)	“I didn’t feel that I was alone in the journey” (P016) “Any worry or advice I needed, I was supported so much” (P056) “Support mentally, should be given to all patients” (P065)
Diet and exercise knowledge	17 (22.1)	“Without it, I would be clueless on what to eat or do in terms of exercise” (P137) “Being aware of different exercises and movements which can be continued long term” (P148)
Confidence to exercise	16 (20.8)	“Gave you the confidence after the surgery to know what exercise was safe to do and to push yourself in a safe environment where you did not feel self-conscious about your loose skin” (P064) “Helped me not to be afraid of exercise” (P139)
Coping with challenges	7 (9.1)	“Fend off temptations. Cope with society and people in general” (P077) “It helped me cope with all the changes” (P045)
Keeping on the right track	6 (7.8)	“Even if you are ultimately accountable for yourself, having [tele-counsellor and exercise therapist names] advice helped to stop me slipping too far in bad months” (P069) “It has helped me watch out for the pitfalls and how to turn it around” (P151)
**Recommendations**	***n*** (%)	**Quotations**
Longer duration of program	7 (77.8)	“Longer than 12 weeks in exercise sessions and maybe start earlier than 3 months” (P019) “I think that this program should be given to after all bariatric surgery and for a longer time to achieve better weight maintenance results.” (P097)
Pre-surgery lifestyle program	2 (22.2)	“I would suggest the program is given to people prior to surgical intervention, if may save the NHS more money in the long run” (P002)

## Discussion

Quantitatively, the tele-counselling program demonstrated high acceptability, reflected in its lower refusal rate, higher attendance rates, and the large proportion of participants who rated the sessions as useful. These findings were further supported by qualitative data, with participants highlighting the benefits of the tele-counselling program in helping them adapt to the lifestyle changes following bariatric surgery. In contrast, the supervised exercise program showed moderate acceptability, as indicated by a higher refusal rate with moderate to high attendance rates. Nevertheless, qualitative findings revealed that participants who attended the sessions consistently found the program valuable in helping them overcome the physical and psychological barriers to exercise that are commonly faced by individuals with overweight and obesity [12]. Key barriers to participation in both interventions included time constraints arising from work demands, family commitments, and scheduling conflicts. To improve the acceptability of future interventions, recommendations include the integration of mobile technology, increasing the frequency of tele-counselling sessions, enhancing accessibility to exercise classes, and providing personalized exercises tailored to an individual’s physical fitness level.

Despite the BARI-LIFESTYLE program not showing a favorable impact on weight loss and health outcomes, participants still acknowledged its benefits. The program was perceived to provide holistic support, encompassing diet, exercise, social, and psychological aspects. A previous qualitative study reported patients experienced a sense of “abandonment” and “isolation” following MBS [13]. They felt they did not receive sufficient support after surgery, with a lack of information and guidance on managing life post-surgery. As a result, the study emphasized the need for effective and acceptable follow-up care packages for bariatric surgery patients in the UK [13]. The present study has provided some approaches that could be considered. Firstly, the high adherence rate to the tele-counselling sessions demonstrated that delivering nutritional-behavioral consultations via telephone is both feasible and acceptable. By reducing the facility and infrastructure costs required for in-person hospital consultations, and improving time efficiency for healthcare professionals, this method enables more frequent post-surgery consultations to be offered to patients. This increased access is crucial, as most participants require additional support once their weight loss begins to plateau. Previous research has shown that better adherence to follow-up care is linked to improved weight loss outcomes [4].

Secondly, to support tele-counselling consultations, the use of mHealth apps for tracking food intake, physical activity, nutritional supplementation, and body weight should be considered, as suggested by participants in the present study. This adoption could facilitate the smooth delivery of remote teleconsultation while minimizing patients’ burden. Indeed, mHealth has shown promise in promoting better weight loss outcomes following MBS [14]. Thirdly, the use of video consultation has also been suggested by participants in the present study, which has been previously utilized in The Bariatric Surgery and Education (BaSE) RCT [15]. The COVID-19 pandemic has revolutionized telemedicine use in healthcare, including weight loss treatment, with videoconferencing being more advantageous in terms of the interpersonal connection associated with in-person care [16]. Therefore, telemedicine should be integrated into the post-bariatric follow-up package with a blended model of delivery [17]. This aligns with the NHS Long Term Plan, which promotes the mainstream use of technology in prevention, care, and treatment across the NHS [18]. Indeed, following the COVID-19 pandemic, the National Institute for Health and Care Excellence (NICE) conducted an early value assessment of digital technologies for delivering multidisciplinary obesity services. As a result, nine digital services were approved to provide digital weight management programs within the NHS [19]. This is in line with recently NICE obesity guidance focused on enhancing access to multidisciplinary weight management services, including for individuals without specialist weight-management services available in their region [19]. However, patients from lower socio-economic backgrounds may experience digital exclusion, which can affect their participation in such programs [17].

The fact that approximately one-fifth of participants in the intervention group did not enroll in the exercise sessions emphasizes the need to address barriers to physical activity after MBS. Consistent with our findings, Possmark et al. reported that patients often prioritized family and work commitments over physical activity [20]. Additionally, some patients did not perceive exercise as necessary or recognize its benefits. This has also been reported by Zabatiero et al. who found some patients considered exercise unnecessary during the first 6 months post-surgery since weight loss will happen regardless [21]. Therefore, efforts should be made to provide continuous patient education from pre- to post-surgery regarding the health benefits of physical activity, particularly in relation to the preservation of muscle and bone mass [22–24]. Another barrier to the uptake and participation in exercise programs is the geographical location of the exercise facility, inconvenient exercise scheduling, and travel time. This has also been reported in a previous post-bariatric exercise RCT [25]. To address this barrier, future studies should consider tele-exercise as an option. We have shown that this method of exercise delivery is feasible and acceptable for patients after MBS [9].

The tailored supervised exercise sessions were perceived to help address the common barriers faced by patients to be physically active following MBS such as poor exercise knowledge and confidence level, fear of injury, physical limitation, and inadequate professional support [21, 26, 27]. Studies have shown that when patients follow a regimen personalized to their functional capacity and fitness level, they are more likely to enjoy the sessions, stay motivated, and remain engaged in physical activity long-term [9]. As our findings suggest, providing exercises that are either too intense or too easy can lead to a loss of interest [9]. If the exercise is too intense, patients may feel overwhelmed or discouraged, whereas if it is too easy, they may not find it sufficiently challenging or beneficial. Striking the right balance by tailoring the program to each patient’s abilities and progress is essential for maintaining engagement in physical activity after bariatric surgery.

This study is not without limitations. The adherence rate, particularly for the supervised exercise program, might have been influenced by the nationwide COVID-19 lockdown. This is despite our efforts to convert the in-person supervised exercise sessions to tele-exercise [9]. Regardless of the efforts of tele-counsellors and exercise therapists, the reasons for rescheduled, cancelled, and missed intervention sessions cannot be fully captured and documented due to poor reporting from participants. Hence, the reasons cannot be specifically quantified. The qualitative data in this study is based on a self-reported exit questionnaire rather than semi-structured interviews or focus groups. Therefore, the in-depth insights into patients’ views and experiences of the program cannot be fully captured. Lastly, the views and experiences of tele-counsellors and exercise therapists delivering the intervention are equally important to consider, as they can provide valuable insights into the challenges, barriers, and facilitators of engagement, helping to refine and enhance the acceptability of future programs. Hence, future studies should consider exploring their perspectives.

## Conclusion

Participant-reported outcomes of the BARI-LIFESTYLE program suggest that it provided holistic support, addressing diet, exercise, social, and psychological aspects of life after bariatric surgery, despite not eliciting additional weight loss or health benefits. This suggests that post-surgical lifestyle programs should not only focus on weight loss outcomes but also on the other wider health benefits gained from participating in these programs.

## Supplementary Information

Below is the link to the electronic supplementary material.

Supplementary file 1 (DOCX 261 KB)

## Data Availability

No datasets were generated or analysed during the current study.

## References

[1] Brown W, Kow L, Anvari M, Ghaferi A, Morton J, Shikora S, et al. 8th IFSO global registry report. InternationaL Federation for Surgery for Obesity and Metabolic Disorders. 2024. Available from: https://www.ifso.com/pdf/8th-ifso-registry-report-2024-latest-new.pdf. Accessed 19 Feb 2025.

[2] Manning S, Pucci A, Carter NC, Elkalaawy M, Querci G, Magno S, et al. Early postoperative weight loss predicts maximal weight loss after sleeve gastrectomy and Roux-en-Y gastric bypass. Surg Endosc. 2015;29(6):1484–91. 10.1007/s00464-014-3829-7. 25239175 10.1007/s00464-014-3829-7 PMC4422859

[3] Sheets CS, Peat CM, Berg KC, White EK, Bocchieri-Ricciardi L, Chen EY, et al. Post-operative psychosocial predictors of outcome in bariatric surgery. Obes Surg. 2015;25(2):330–45. 10.1007/s11695-014-1490-9. 25381119 10.1007/s11695-014-1490-9 PMC4538993

[4] Hood MM, Corsica J, Bradley L, Wilson R, Chirinos DA, Vivo A. Managing severe obesity: understanding and improving treatment adherence in bariatric surgery. J Behav Med. 2016;39(6):1092–103. 10.1007/s10865-016-9772-4. 27444752 10.1007/s10865-016-9772-4

[5] National Institute for Health and Care Excellence (NICE). Obesity: identification, assessment and management. 2014. Available from: https://www.nice.org.uk/guidance/cg189/chapter/1-recommendations. Accessed 19 Feb 2025. 36719951

[6] Jassil FC, Carnemolla A, Kingett H, Doyle J, Kirk A, Lewis N, et al. Impact of nutritional-behavioral and supervised exercise intervention following bariatric surgery: the BARI-LIFESTYLE randomized controlled trial. Obesity (Silver Spring). 2023;31(8):2031–42. 10.1002/oby.23814. 37415246 10.1002/oby.23814

[7] Baillot A, St-Pierre M, Bernard P, Burkhardt L, Chorfi W, Oppert JM, et al. Exercise and bariatric surgery: a systematic review and meta-analysis of the feasibility and acceptability of exercise and controlled trial methods. Obes Rev. 2022;23(9):e13480. 10.1111/obr.13480. 35695385 10.1111/obr.13480

[8] Jassil FC, Carnemolla A, Kingett H, Paton B, O’Keeffe AG, Doyle J, et al. Protocol for a 1-year prospective, longitudinal cohort study of patients undergoing Roux-en-Y gastric bypass and sleeve gastrectomy: the BARI-LIFESTYLE observational study. BMJ Open. 2018;8(3):e020659. 10.1136/bmjopen-2017-020659. 29549212 10.1136/bmjopen-2017-020659 PMC5857659

[9] Jassil FC, Richards R, Carnemolla A, Lewis N, Montagut-Pino G, Kingett H, et al. Patients’ views and experiences of live supervised tele-exercise classes following bariatric surgery during the COVID-19 pandemic: the BARI-LIFESTYLE qualitative study. Clin Obes. 2022;12(2):e12499. 10.1111/cob.12499. 34841676 10.1111/cob.12499 PMC9011650

[10] Jassil FC, Papageorgiou M, Mackay E, Carnemolla A, Kingett H, Doyle J, et al. One-year changes in body composition and musculoskeletal health following metabolic/bariatric surgery. J Clin Endocrinol Metab. 2024. 10.1210/clinem/dgae496. 10.1210/clinem/dgae496 PMC12012674 39108088

[11] Krippendorff K. Content analysis: an introduction to its methodology. Thousand Oaks, California: SAGE Publications, Inc.; 2019. Available from: https://methods.sagepub.com/book/mono/content-analysis-4e/toc. Accessed 19 Feb 2025.

[12] McIntosh T, Hunter DJ, Royce S. Barriers to physical activity in obese adults: a rapid evidence assessment. J Res Nurs. 2016;21(4):271–87. 10.1177/1744987116647762.

[13] Coulman KD, MacKichan F, Blazeby JM, Donovan JL, Owen-Smith A. Patients’ experiences of life after bariatric surgery and follow-up care: a qualitative study. BMJ Open. 2020;10(2):e035013. 10.1136/bmjopen-2019-035013. 32034030 10.1136/bmjopen-2019-035013 PMC7045271

[14] Mangieri CW, Johnson RJ, Sweeney LB, Choi YU, Wood JC. Mobile health applications enhance weight loss efficacy following bariatric surgery. Obes Res Clin Pract. 2019;13(2):176–9. 10.1016/j.orcp.2019.01.004. 30826256 10.1016/j.orcp.2019.01.004

[15] Wild B, Hunnemeyer K, Sauer H, Hain B, Mack I, Schellberg D, et al. A 1-year videoconferencing-based psychoeducational group intervention following bariatric surgery: results of a randomized controlled study. Surg Obes Relat Dis. 2015;11(6):1349–60. 10.1016/j.soard.2015.05.018. 26421929 10.1016/j.soard.2015.05.018

[16] Ufholz K, Bhargava D. A review of telemedicine interventions for weight loss. Curr Cardiovasc Risk Rep. 2021;15(9):17. 10.1007/s12170-021-00680-w 10.1007/s12170-021-00680-w PMC8280385 34306296

[17] Brown A, Louisa E, Stuart F, Jamie B, Alison F, Lisa M, et al. Supporting weight management service during the COVID-19 pandemic: phase I insights. 2020. Available from: https://assets.publishing.service.gov.uk/media/5f55f57dd3bf7f4d6eb12eb9/WMS_Report.pdf. Accessed 19 Feb 2025.

[18] National Health Service. The NHS long term plan. Chapter 5: Digitally-enabled care will go mainstream across the NHS. 2019. Available from: https://www.longtermplan.nhs.uk/online-version/chapter-5-digitally-enabled-care-will-go-mainstream-across-the-nhs/. Accessed 19 Feb 2025.

[19] National Institute for Health and Care Excellence (NICE). Digital technologies for delivering multidisciplinary weight-management services: early value assessment. NICE Health Technology Evaluation HTE14. 2024. Available from: https://www.nice.org.uk/guidance/hte14. Accessed 19 Feb 2025.

[20] Possmark S, Berglind D, Sellberg F, Ghaderi A, Persson M. To be or not to be active - a matter of attitudes and social support? Women’s perceptions of physical activity five years after Roux-en-Y gastric bypass surgery. Int J Qual Stud Health Well-being. 2019;14(1):1612704. 10.1080/17482631.2019.1612704. 31072238 10.1080/17482631.2019.1612704 PMC6522969

[21] Zabatiero J, Smith A, Hill K, Hamdorf JM, Taylor SF, Hagger MS, et al. Do factors related to participation in physical activity change following restrictive bariatric surgery? A qualitative study. Obes Res Clin Pract. 2018;12(3):307–16. 10.1016/j.orcp.2017.11.001. 29150223 10.1016/j.orcp.2017.11.001

[22] Gil S, Kirwan JP, Murai IH, Dantas WS, Merege-Filho CAA, Ghosh S, et al. A randomized clinical trial on the effects of exercise on muscle remodelling following bariatric surgery. J Cachexia Sarcopenia Muscle. 2021;12(6):1440–55. 10.1002/jcsm.12815. 34666419 10.1002/jcsm.12815 PMC8718087

[23] Murai IH, Roschel H, Dantas WS, Gil S, Merege-Filho C, de Cleva R, et al. Exercise mitigates bone loss in women with severe obesity after Roux-en-Y gastric bypass: a randomized controlled trial. J Clin Endocrinol Metab. 2019;104(10):4639–50. 10.1210/jc.2019-00074. 31322672 10.1210/jc.2019-00074

[24] Diniz-Sousa F, Veras L, Boppre G, Sa-Couto P, Devezas V, Santos-Sousa H, et al. The effect of an exercise intervention program on bone health after bariatric surgery: a randomized controlled trial. J Bone Miner Res. 2021;36(3):489–99. 10.1002/jbmr.4213. 33295063 10.1002/jbmr.4213

[25] Mundbjerg LH, Stolberg CR, Cecere S, Bladbjerg EM, Funch-Jensen P, Gram B, et al. Supervised physical training improves weight loss after Roux-en-Y gastric bypass surgery: a randomized controlled trial. Obesity (Silver Spring). 2018;26(5):828–37. 10.1002/oby.22143. 29566463 10.1002/oby.22143

[26] Wiklund M, Olsen MF, Olbers T, Willen C. Experiences of physical activity one year after bariatric surgery. Open Obes J. 2014;6:25–30.

[27] Peacock JC, Sloan SS, Cripps B. A qualitative analysis of bariatric patients’ post-surgical barriers to exercise. Obes Surg. 2014;24(2):292–8. 10.1007/s11695-013-1088-7. 24092517 10.1007/s11695-013-1088-7

