# Supplementary material for: Exploring the Acceptability of Post-bariatric Nutritional-Behavioral and Supervised Exercise Intervention (BARI-LIFESTYLE): A Mixed Methods Evaluation
Source: Obes Surg. 2025 May 31;35(7):2471–9. doi: 10.1007/s11695-025-07927-0 (PMC12271284; doi:10.1007/s11695-025-07927-0)
Supplement: Supplementary file 1 — (DOCX 261 KB) [file 11695_2025_7927_MOESM1_ESM.docx]

**Exploring the acceptability of post-bariatric nutritional-behavioral and supervised exercise intervention (BARI-LIFESTYLE): a mixed methods evaluation**

Table of Contents

[Supplementary S1: Example of food diary in the tele-counselling booklet 2](#_Toc191738677)

[Supplementary S2: Example of exercise diary in the exercise booklet 4](#_Toc191738678)

[Supplementary S3: Exit questionnaire 6](#_Toc191738679)

[Supplementary S4: Exit questionnaire results 10](#_Toc191738680)

# Supplementary S1: Example of food diary in the tele-counselling booklet

**ONE DAY DIARY FOR TELE-COUNSELLING (SESSION 6)**

| **Day of the week** | **Weight (kg) Once per week** | **Fitbit steps** | **Supplements** | | | **What I ate/ Drank** | **Protein**  **3 portions daily** | **Carbs**  **2-3 portions daily** | **Milk and dairy**  **2-3 portions daily** | **Fruit & veg 5 portions daily** | **Fats/ sugars** | **2L calorie free liquid** |
| --- | --- | --- | --- | --- | --- | --- | --- | --- | --- | --- | --- | --- |
|  |  |  | **Multi-**  **vitamin**  **once daily** | **Iron once daily** | **Calcium and Vit D twice daily** |  |  |  |  |  |  |  |
|  | Enter weight below | Enter steps below | Please tick (🗸) below | | |  | Please circle the appropriate number | | | | Please comment if consumed | Please specify below |
|  |  |  |  |  |  |  | 1  2  3 | 1  2  3 | 1  2  3 | 1  2  3 |  |  |
| **Total number of servings** | | | | | | |  |  |  |  |  |  |

**NOTES (SESSION 6)**

# Supplementary S2: Example of exercise diary in the exercise booklet

# Supplementary S3: Exit questionnaire

**
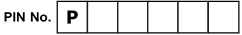
**
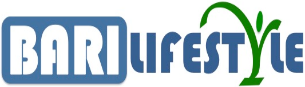


**FEEDBACK FORM FOR THE BARI-LIFESTYLE INTERVENTION STUDY**

Choose one option for each questionnaire item.

| **EVALUATION OF THE TELE-COUNSELLING SESSIONS** |
| --- |
| 1. How useful did you find the tele-counselling sessions?   Very useful  Useful  Neither  Useless  It was a waste of time |
| 1. Did you find any topic particularly interesting/ useful?   Yes If yes, please specify: __________________________________________________  No |
| 1. Were you satisfied with the length of the calls? (tick all that apply)   Yes  No  Too long  Too short  About right |
| 1. Were you satisfied with the quality of the diaries and study material provided?   Very satisfied  Satisfied  Neither  Unsatisfied  Very unsatisfied |
| 1. How easy was it for you to use the dairies/ study material?   Very easy  Easy  Neither  Difficult  Very difficult  **Continued on next page** |

| 1. How satisfied were you with the help provided by the research team on how to complete the diaries/ use the study material (if applicable)?   Very satisfied  Satisfied  Neither  Unsatisfied  Very unsatisfied  Not applicable |
| --- |
| 1. Did you have any difficulty in booking the sessions for a time that was convenient to you?   Yes If yes, please specify: __________________________________________________  No |
| 1. Would you suggest we make any changes in the tele-counselling programme?   Yes If yes, please specify: __________________________________________________  No |

| **EVALUATION OF THE SUPERVISED EXERCISE PROGRAMME** |
| --- |
| 1. How useful did you find the supervised exercise programme?   Very useful  Useful  Neither  Useless  It was a waste of time |
| 1. What exercise did you enjoyed most?   __________________________________________________________________________________________________________________________________________________________ |
| 1. What exercise did you enjoyed least?   __________________________________________________________________________________________________________________________________________________________ |
| 1. What exercise did you find particularly difficult?   __________________________________________________________________________________________________________________________________________________________  **Continued on next page** |

| 1. Were you satisfied with the length of the supervised exercise sessions? (tick all that apply)   Yes  No  Too long  Too short  About right |
| --- |
| 1. Were you satisfied with the quality of time the physiotherapist dedicated to you?   Yes  No  Comments: ___________________________________________________________________ |
| 1. Were you satisfied with the amount of time the physiotherapist dedicated to you?   Yes  No  Comments: ___________________________________________________________________ |
| 1. Were you satisfied with the study material provided to you for the home exercise sessions?   Yes  No  Comments: ___________________________________________________________________ |
| 1. Did you have any difficulty in booking the exercise sessions for a time that was convenient to you?   Yes If yes, please specify: __________________________________________________  No |
| 1. Would you suggest we make any changes in the supervised exercise programme?   Yes If yes, please specify: __________________________________________________  No |

| **FINAL OVERVIEW** |
| --- |
| 1. Do you think that participating in this programme helped you cope with the lifestyle changes needed to adapt after bariatric surgery?   Yes If yes, in what way : ______________________________________________  ______________________________________________  No If not, why : ­­­­­­­­­­­­­______________________________________________  ______________________________________________  **Continued on next page** |
| 1. Do you have any suggestion that could help us improve this programme in the future?   ___________________________________________________________________________________________________________________________________________________________________________________________________________________________________________________________________________________________________________________________________________________________________________________________________________________________________________________________________________________________________________________________________________________________ |

**END OF QUESTIONNAIRE – THANK YOU**

# Supplementary S4: Exit questionnaire results

| **Tele-counselling programme (*n*=63)** | ***n* (%)** |
| --- | --- |
| How useful did you find the tele-counselling sessions?  Very useful  Useful  Neither  Useless  It was a waste of time | 48 (76.2)  13 (20.6)  2 (3.2)  0  0 |
| Were you satisfied with the length of the calls?  Yes  No  Too long  Too short  About right | 53 (84.1)  0  0  1 (1.6)  9 (14.3) |
| Were you satisfied with the quality of the diaries and study material provided?  Very satisfied  Satisfied  Neither  Unsatisfied  Very unsatisfied | 41 (65.1)  15 (23.8)  5 (7.9)  2 (3.2)  0 |
| How easy was it for you to use the dairies/ study material?  Very easy  Easy  Neither  Difficult  Very difficult | 38 (60.3)  18 (28.6)  2 (3.2)  5 (7.9)  0 |
| How satisfied were you with the help provided by the research team on how to complete the diaries/ use the study material?  Very satisfied  Satisfied  Neither  Unsatisfied  Very unsatisfied  Not applicable | 47 (74.6)  10 (15.9)  6 (9.5)  0  0  0 |
| Did you find any difficulty in booking the sessions for a time that was convenient to you?  Yes  No  Did not answer | 4 (6.3)  58 (92.1)  1 (1.6) |
| **Supervised Exercise Programme (*n*=52)** | ***n* (%)** |
| How useful did you find the supervised exercise programme?  Very useful  Useful  Neither  Useless  It was a waste of time | 44 (84.6)  7 (13.5)  1 (1.9)  0  0 |
| Were you satisfied with the length of the supervised exercise sessions?  Yes  No  Too long  Too short  About right | 36 (69.2)  0  1 (1.9)  7 (13.5)  8 (15.4) |
| Were you satisfied with the quality of time the physiotherapist dedicated to you?  Yes  No  Did not answer | 48 (92.3)  3 (5.8)  1 (1.9) |
| Were you satisfied with the amount of time the physiotherapist dedicated to you?  Yes  No  Did not answer | 46 (88.5)  4 (7.7)  2 (3.8) |
| Were you satisfied with the study material provided to you for the home exercise sessions?  Yes  No  Did not answer | 49 (94.2)  2 (3.9)  1 (1.9) |
| Did you have any difficulty in booking the exercise sessions for a time that was convenient to you?  Yes  No  Did not answer | 6 (11.6)  45 (86.5)  1 (1.9) |
| Would you suggest we make any changes in the supervised exercise programme?  Yes  No | 14 (26.9)  38 (73.1) |
| **Final overview (*n*=63)** |  |
| Do you think that participating in this programme helped you cope with the lifestyle changes needed to adapt after bariatric surgery?  Yes  No | 63 (100)  0 |
